# A solution for the rational dispatching of concrete transport vehicles

**DOI:** 10.1038/s41598-022-21011-y

**Published:** 2022-10-06

**Authors:** Zhi-guang Guo, Yong-fu Liu, Chang-jiang Ao

**Affiliations:** China Construction Civil Engineering Co., Ltd., Beijing, 100073 China

**Keywords:** Civil engineering, Energy infrastructure

## Abstract

The amount of concrete needed in the construction of projects is enormous. The concrete dispatching in construction is, however, chaotic and causes grievous waste due to the tight construction schedule, heavy tasks, and improper transport vehicle dispatching by the constructor. This paper proposes a more realistic objective function in the vehicle dispatching algorithm and offers a solution to the rational dispatching of concrete transport vehicles. The analysis of the calculation example validates the feasibility of the built model, which provides more appropriate dispatching and more balanced distribution, meets the needs of the worksite and mixing station to the greatest extent and improves the level of project refinement and process management. Therefore, the model is worth promoting in future practice.

## Introduction

The amount of concrete needed in the construction of projects is enormous, requiring transportation of concrete from the mixing station to the construction site by trucks. The concrete dispatching in construction is, however, chaotic and causes grievous waste due to the tight construction schedule, heavy tasks, and improper transport vehicle dispatching by the constructor^1–4^. Hence, it is necessary to develop a proper optimization plan to realize trucks' rational dispatching conveniently.

Mathematically, the dispatching of trucks is a mixed-integer. It is a programming problem, which constitutes an NP-hard problem academically that is difficult to solve. Bionic algorithms that imitate various ecosystems in nature can get better results in solving many complex optimization problems, which are practical, versatile, flexible, and efficient. Therefore, bionic algorithms have become an important direction and research hotspot for solving optimization problems in recent years^5,6^.

Kennedy and Eberhart, inspired by the foraging behavior of birds in nature, proposed a particle swarm optimization (PSO) algorithm in 1995^7^. Because of its simple structure and fast convergence, PSO is widely used to solve optimization problems. There have been numerous modified PSO algorithms focusing on different aspects of the algorithm by far. The standard PSO algorithm (PSO-S) is as follows^8–11^:

1 $${\varvec{v}}_{p}^{t + 1} = \omega {\varvec{v}}_{p}^{t} + c_{1} {\varvec{r}}_{1} \left( {{\varvec{pbest}}_{p}^{t} - {\varvec{x}}_{i}^{t} } \right) + c_{2} {\varvec{r}}_{2} \left( {{\varvec{gbest}}^{t} - {\varvec{x}}_{p}^{t} } \right)$$

2 $${\mathbf{x}}_{p}^{t + 1} = {\mathbf{x}}_{p}^{t} + {\mathbf{v}}_{p}^{t + 1}$$

where, *p* = 1, 2, … *N* is the population size; *w* is the weight of inertia; ${{\varvec{v}}}_{p}^{t}$ and ${{\varvec{x}}}_{p}^{t}$ are the velocity and position and direction vectors of particle *p* in the *t*-th generation; ${{\varvec{p}}{\varvec{b}}{\varvec{e}}{\varvec{s}}{\varvec{t}}}_{p}^{t}$ and ${{\varvec{g}}{\varvec{b}}{\varvec{e}}{\varvec{s}}{\varvec{t}}}^{{\varvec{t}}}$ are the individual optimal of particle *p* of the *t*-th generation and the global optimal of all particles; *c*_*1*_ and *c*_*2*_ are the individual perceived weights and the overall social weights; *r*_*1*_ and *r*_*2*_ are two random *D*-dimensional vector parameters evenly distributed in [0, 1].

Scholars have paid attention to vehicle dispatching. Javier et al.^1^ introduced the balanced dispatching problem in passengers transport services (BDP-PTS) on demand, which seeks a dispatching solution that aims to minimize the variance of the incomes per unit of working time among the drivers. In addition, computational experiments are carried out to compare the proposed online dispatching algorithms and the MIQP model on datasets of real complete instances from a Chilean transport company. Chen et al.^2^ proposed an approach that allows data-driven and schedule-oriented supply chain coordination in the face of demand fluctuations, and demonstrated the coordination approach through an example project featuring a 5-day in-situ construction of concrete walls. Lin et al.^3^ described the scheduling operation of ready mixed concrete (RMC) trucks as a circulating job shop problem in a multi-objective programming model, and points out that the total waiting time from the mixing station shall not exceed 1.5 h to maintain the concrete quality during the driving of the ready mixed transport truck. Liu et al.^4^ focused on the integration of ready mixed concrete production scheduling and truck and pump scheduling in a ready mixed concrete (RMC) plant with multiple mixers, and proposed a spatiotemporal network model that combines RMC production and vehicle scheduling. Ostroukh et al.^12^ proposed the scientific approach to the problem for automation transportation planning construction materials in the two-tier control system. Yan et al.^13^ employed problem decomposition and relaxation techniques, with a mathematical programing solver, to develop an algorithm that is capable of efficiently solving the problem. Aleksander^14^ presented a model for the selection of optimal transportation system inside the precast concrete manufacturing plant, which ensures that the fullest possible use of equipment resources, in this case, rail trolleys capacity, and minimization of their journey number. Srichandum and Rujirayanyong^15^ built a model based on bee colony optimization (BCO) to find the best dispatching plan that minimizes the total waiting time of ready mixed concrete (RMC) trucks on the construction site. Zeng et al.^16^ based on the reverse method of PSO, solved the queuing network problem with fuzzy data in a specific transport system. Some scholars focused on the opportunity constraints in concrete vehicle dispatching^17^, fuel cost minimization dispatching^18^, and entrusting MAS to solve the constraint problems^19^. Kim et al.^20^ proposed a dynamic model for PC production scheduling by adopting a discrete-time simulation method to respond to due date changes in real time and by using a new dispatching rule that considers the uncertainty of the due dates to minimize tardiness. Rau et al.^21^ proposed a discrete multi swarm particle swarm optimization (PSO) algorithm and a heuristic optimization algorithm to generate the Pareto set of mocgirp, and applied various sensitivity analysis settings of multiple parameters (available vehicles, vehicle capacity, vehicle fuel efficiency, warehouse capacity and warehouse energy consumption) to MT and St routing methods. Some methods consider the influence of some influencing factors to the objective function considers and some consider minimal constraints. However, their applicability is poor, such as order modification and vehicle failure are not considered, and the problem of minimizing the waiting time of vehicles and mixing stations is not fully considered, which is often important for the construction site.

This study analyzed each influencing factor in detail, proposed a more realistic objective function in the dispatching algorithm and offered a solution to the rational dispatching of trucks for different waiting times based on actual problem requirements. Meanwhile, it described and improved the initialization and iterative process in the calculation process in detail. It also provided solutions to the dynamic changes of temporarily increasing or canceling orders and increasing or decreasing the number of available trucks.

## Algorithm model optimization

### Modeling

Combined with the problem of truck dispatching, the worksite is selected as the updated position of the particle swarm (*x*). Then, the concrete demand sites were numbered sequentially (1, 2, 3, … *x*_*max*_). By counting the total demand for concrete, the total number of departures (*N*_*gc*_) required to complete the task was calculated. Then, the solution to this problem has *N*_*gc*_-dimensional coordinates; namely, each particle *p* corresponds to *N*_*gc*_-dimensional data. For example:

3 $${\mathbf{x}}_{p} = \left[ {x_{1} ,x_{2} , \ldots x_{{N_{{{\text{gc}}}} }} } \right]$$

where, ${x}_{1}$ is the sequence number of the worksite selected for the first departure, and ${x}_{{N}_{\text{gc}}}$ is the sequence number of the worksite selected for the last departure.

Since ***x*** is only an integer, the particle velocity should also be taken as an integer, and Eq. (1) is modified as follows:

4 $${\mathbf{v}}_{p}^{t + 1} = \left\langle {\omega {\mathbf{v}}_{p}^{t} + c_{1} {\mathbf{r}}_{1} \left( {{\mathbf{pbest}}_{p}^{t} - {\mathbf{x}}_{p}^{t} } \right) + c_{2} {\mathbf{r}}_{2} \left( {{\mathbf{gbest}}^{t} - {\mathbf{x}}_{p}^{t} } \right)} \right\rangle$$

where, 〈 〉 represents an integer after rounding. The value range of velocity is an integer in [$-{x}_{\text{max}}$, ${x}_{\text{max}}$], and ${x}_{\text{max}}$ is the maximum value for the sequence number of the worksite. When the velocity exceeds the boundary, the boundary value shall be taken.

In PSO algorithms, the inertia weight *w* reflects the global searchability. When it is larger, the global searchability is stronger; conversely, the local searchability is more potent^22–24^. Generally, in the initial stage of iterative calculation, it is hoped that the search range of particles is wider; and in the later stage of the search, it is hoped that particles can search accurately within a smaller range. In addition, the inertia weights of different particles should be different under the same number of iterations to enhance the searchability of the particles. Therefore, the inertia weight can be calculated as follows:

5 $$w = w_{\max } - \frac{{\left( {w_{\max } - w_{\min } } \right) \cdot t}}{{t_{\max } }} + \frac{Rnd}{3}$$

where, ${w}_{\text{max}}$ is the maximum value of *w*, and 0.9 is recommended; ${w}_{\text{min}}$ is the minimum value of w*,* and 0.4 is recommended; ${t}_{\text{max}}$ represents the maximum number of iterations; *t* is the number of iterations of the current calculation step; R*nd* represents a random number in [0,1].

Equation (4) can be used to obtain the corresponding velocity value, and according to Eq. (2), the updated position of the particle, that is, the worksite number selected by the truck, can be calculated. When the updated particle position crosses the boundary (i.e., *x* < 1 or *x* > ${x}_{\text{max}}$), a worksite is randomly selected until a position that does not cross the boundary is found. Calculations can be performed as follows:

6 $$x_{p} = \left\{ {\begin{array}{*{20}l} {\left\langle {Rnd \cdot x_{\max } + 0.5} \right\rangle ;} \hfill & {\left( {x < 1} \right) \vee \left( {x > x_{\max } } \right)} \hfill \\ {x;} \hfill & {1 \le x \le x_{\max } } \hfill \\ \end{array} } \right.$$

where, “$\vee$” represents an “or” set; “;” represents the function is a piecewise function, when the conditions after the semicolon are satisfied, the function takes the value before the semicolon.

Furthermore, since the completion time of pour at different worksites varies, the particles are likely to select the worksites that have completed the pour in the iterative process, which will produce too many invalid solutions, increase the amount of calculation, and reduce the convergence. Therefore, based on bionic algorithms, the sequence number of the selected worksite is increased by 1 (*v*_*p*_ > 0) or decreased by 1 (*v*_*p*_ < 0) for the location of the selected worksites which have completed the pour according to the positive or negative velocity, until there is a worksite that has not completed the pour. In the process, when the worksite selected is out of bounds, take the symmetric boundary value: when *x*_*p*_ > ${x}_{\text{max}}$ and *v*_*p*_ > 0, then *x* = 1; similarly, when *x*_*p*_ < 1 and *v*_*p*_ < 0, then *x* = ${x}_{\text{max}}$.

### Initialization (first iteration)

The velocity of the first dimensional coordinate of each particle *p* (i.e., the first departure in the solution) is 0. To avoid the initial velocity from being too large and resulting in a local optimum, the velocity of the coordinates in the remaining dimensions is calculated as follows, taking an integer in [$-2\cdot {x}_{\text{max}}/3$, $2\cdot {x}_{\text{max}}/3$].

7 $$v_{p} = \left\langle {\frac{{2 \cdot \left( {2 \cdot Rnd - 1} \right)}}{3} \cdot x_{\max } } \right\rangle$$

Except for the first dimensional coordinate of each particle *p*, the working points of the other-dimensional coordinates are randomly selected and calculated as follows:

8 $$x_{p} = \left\langle {Rnd \cdot x_{\max } + 0.5} \right\rangle$$

To avoid too many locally optimal solutions and invalid solutions, the sequence number of the first dimensional coordinates of the particle is determined according to the start time of each worksite and the cycle time of completing a pour task. Obviously, when the number of the earliest worksite is one, to ensure that the worksite is started in time, it is considered as the worksite of the first dimensional coordinate of the particle; when the number of the earliest worksites is more than one, to ensure the maximum utilization of the truck, the worksite with the shortest cycle time is selected as the worksite of the first dimensional coordinates of the particles.

### Iterative process

As the worksite for the first departure has been determined according to the start time of each worksite and the cycle time of completing a pour task, the velocity of the first dimensional coordinate of each particle (i.e., the first departure in the solution) is 0; the velocity of the other dimensions is calculated according to Eq. (4). When the velocity exceeds the boundary, take the boundary value.

The worksite of particles is calculated according to Eq. (2), and when the position is out of bounds, it is calculated according to Eq. (6). Similar to the procedure described in the Initialization section, if the worksite has completed the pour, the sequence number of the selected worksite is increased or decreased by 1 according to the positive or negative velocity, until there is a worksite that has not completed the pour.

### Determination of the objective function

For dispatching problems, the objective function usually takes the waiting time; that is, the shorter the waiting time, the better the solution. For the engineering dispatching problem to be solved in this paper, generally, eight waiting times need to be considered. First, there are two waiting times after each truck has been poured at worksite *i*, namely, the waiting time of the worksite when the *n*-th truck leaves (${TDgd}_{i,n}$) and the waiting time of the truck (${TDgc}_{i,n}$). There are two cumulative waiting times of the worksite *i* after the pour task is completed, namely, the total waiting time of worksite (${Tgd}_{i}$) and the total waiting time of truck (${Tgc}_{i}$). Then, there is a longest total waiting time for any worksites (${TMgd}_{i}$) and trucks (${TMgc}_{i}$). Correspondingly, there are two total cumulative waiting times, namely, the total cumulative waiting time of all worksites (${TS}_{\text{gd}}$) and trucks (${TS}_{\text{gc}}$). They can be calculated as follows:

9 $$\begin{aligned} & Tgd_{i} = \sum\limits_{n = 1}^{{NSVgd_{i} }} {TDgd_{i,n} } ,Tgd_{i} = \sum\limits_{n = 1}^{{NSVgd_{i} }} {TDgc_{i,n} } {\kern 1pt} \\ & TMgd_{i} = \max \left\{ {TDgd_{i,1} ,TDgd_{i,2} , \ldots TDgd_{i,n} , \ldots } \right\} \\ & TMgc_{i} = \max \left\{ {TDgc_{i,1} ,TDgc_{i,2} , \ldots TDgc_{i,n} , \ldots } \right\} \\ & TS_{{{\text{gd}}}} = \sum\limits_{i = 1}^{n} {Tgd_{i} } ,TS_{{{\text{gc}}}} = \sum\limits_{i = 1}^{n} {Tgc_{i} } \\ \end{aligned}$$

where, ${NSVgd}_{i}$ refers to the total number of trucks needed at worksite *i*.

Taking worksite waiting for trucks as an example, according to the actual situation of the project, it is assumed that there are 3 worksites, and worksites A, B, and C require 3, 4, and 5 trucks to complete the pour task, respectively. See Table 1 for details. In addition, the situation of truck waiting for worksite is the same.

**Table 1:** Waiting time corresponding to different schemes (unit: min).

Schemes	worksites	${TDgd}_{i,n}$					${TMgd}_{i}$	${Tgd}_{i}$	${TS}_{\text{gd}}$
		1	2	3	4	5			
I	A	30	10	20	–	–	30	60	230
	B	20	30	20	10	–	30	80	
	C	10	20	20	20	20	20	90	
II	A	40	10	10	–	–	40	60	230
	B	20	20	20	20	–	20	80	
	C	10	20	20	20	20	20	90	
III	A	30	20	20	–	–	30	70	250
	B	20	20	20	20	–	20	80	
	C	20	20	20	20	20	20	100	
IV	A	30	20	20	–	–	30	70	240
	B	20	20	20	20	–	20	80	
	C	10	20	20	20	20	20	90	

Judging from the Table 1, the shortest ${TS}_{\text{gd}}$ of all the worksites is found in plans I and II, and the ${Tgd}_{i}$ of worksites A, B, and C in the two plans is the same. The ${TMgd}_{i}$ of worksite C are also the same. In the two plans, comparing the waiting time of each departure (${TDgd}_{i,n}$) at worksite A, the number in plan I is more even, and there is no extreme long or short waiting time. In this case, plan I is better than plan II. Similarly, comparing the ${TDgd}_{i,n}$ at worksite B, the level is more even in plan II, so plan II is better. From the above analysis, it is not easy to judge which plan is better. Nonetheless, comparing the ${TMgd}_{i}$ of each worksite in the two plans, plan I is more even, which can avoid the longer or shorter waiting time of trucks at some worksites in the plan. In this term, plan I is better than plan II. In summary, the more even the ${TMgd}_{i}$ of all worksites, the better the plan (that is, the ${TMgd}_{i}$ of any plans is not greater than those in other plans).

Comparing the ${TMgd}_{i}$ of each worksite in plans I and III, it is evident that plan III is better than plan I. The ${Tgd}_{i}$ of worksites A and C in plan III is, however, longer than that in plan I, meaning that this plan III is not optimal, and the ${TS}_{\text{gd}}$ is not optimal. Therefore, the ${Tgd}_{i}$ and the ${TS}_{\text{gd}}$ cannot be regarded as the main controlling factors of the objective function; instead, the ${TMgd}_{i}$ should be deemed the main controlling factor.

In addition, the ${TMgd}_{i}$ of worksites in plans III and IV is the same. The ${Tgd}_{i}$ of worksite C in plan IV is shorter, and the ${TS}_{\text{gd}}$ is also shorter. In this sense, plan IV is better than plan III. Therefore, the objective function needs to consider the influence of the ${TS}_{\text{gd}}$ and each worksite's ${Tgd}_{i}$. Generally speaking, while ensuring that the ${TMgd}_{i}$ of the worksite is optimal, the ${TS}_{\text{gd}}$ is directly proportional to the ${Tgd}_{i}$. To simplify the calculation of the objective function, only the ${TS}_{\text{gd}}$ is used as the controlling factor of the objective function.

In terms of the ${TMgd}_{i}$, plan IV is significantly better than plan I, while the ${TS}_{\text{gd}}$ of plan IV is longer than that of plan I. Therefore, the ${TS}_{\text{gd}}$ needs to be revised when considered. For engineering projects, due to the construction schedule and project quality requirements, the waiting time of the worksite generally does not exceed 60 min, and the waiting time of the truck generally ranges from 120 to 150 min as restricted by the initial setting time of the concrete. The waiting time of the worksite is more critical than the waiting time of the truck in this problem, and the main controlling factor of the ${TMgd}_{i}$ has ensured that the waiting time of the truck will not be too long. Hence, only the worksite's ${TS}_{\text{gd}}$ is considered an influencing factor of the objective function, which can fully satisfy the requirements.

In summary, the objective function needs to satisfy the following requirements: the ${TMgd}_{i}$ and ${TMgc}_{i}$ is not longer than that of other plans, and the ${TS}_{\text{gd}}$ is less than 1.2 times that of other plans where 1.2 is the correction coefficient. As mentioned earlier, it is to ensure that all possible optimal solutions are taken into account in the solution set.

Therefore, for each particle, the individual optimal solution of particle *p* can be obtained through the Algorithm 1.
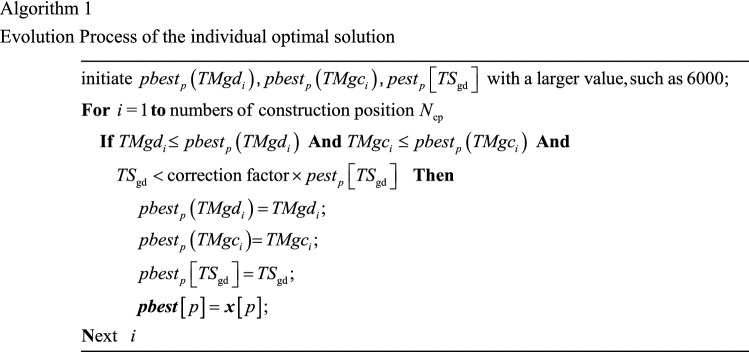


The globally optimal solution can be obtained through the Algorithm 2 for the entire particle swarm.
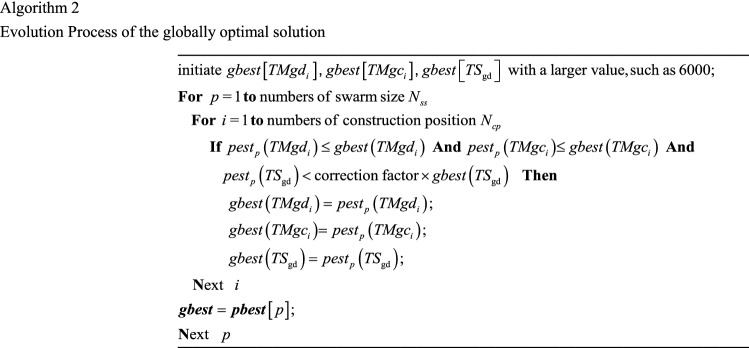


## Solutions to static dispatching

### Departure time of the first truck

To ensure the maximum utilization of trucks and avoid affecting the starting time of the worksite, it is necessary to comprehensively consider factors such as the starting time of the mixing station, the loading and unloading time of the truck, the starting time of the worksite, the number of trucks required, and the time required for a truck cycle to determine the departure time of the first truck (${TI}_{1}$).

Usually, if the truck arrives at the worksite later than the starting time of the worksite, the departure is carried out according to the usual starting time of the mixing station; otherwise, the departure will be determined according to the starting time of the worksite. When there are multiple earliest starting worksites and second earliest starting worksites, the ${TI}_{1}$ should be moved forward by $\Delta t$ to ensure a simultaneous start and minimize waste of truck resources. Moreover, since the initial setting time of concrete is limited, it is necessary to consider the maximum amount of time that the truck can wait to avoid affecting the quality of the concrete. The ${TI}_{1}$ can be calculated as follows:

10 $$TI_{1} = \left\{ {\begin{array}{*{20}l} {T_{{{\text{bhz}}}} ;} \hfill & {TC - \left( {T_{{{\text{bhz}}}} + t_{{{\text{zc}}}} + t_{{{\text{ar}}}} } \right) - \Delta t \le 0} \hfill \\ {TC - \left( {T_{{{\text{bhz}}}} + t_{{{\text{zc}}}} + t_{{{\text{ar}}}} } \right) - \Delta t;} \hfill & {TC - \left( {T_{{{\text{bhz}}}} + t_{{{\text{zc}}}} + t_{{{\text{ar}}}} } \right) - \Delta t > 0} \hfill \\ \end{array} } \right.$$

where, *T*_bhz_ is the starting time of the mixing station; *TC* is the starting time of the earliest worksite; *t*_zc_ is the time required to load the concrete; *t*_ar_ is the shortest time required for a single cycle of the truck at the earliest worksite; $\Delta t$ is time difference corrected by factors such as the time and number of earliest starting worksites and second earliest starting worksites and the maximum waiting time of trucks.

If only one worksite requires concrete, it can be calculated according to Eq. (10), and $\Delta t$ = 0. If there are multiple earliest starting worksites and second earliest starting worksites, the destination of the first truck should be the worksite with the shortest *t*_ar_. Then, it is necessary to determine the $\Delta t$ based on the total number of trucks available in the mixing station (*NSV*_bhz_), the total number of trucks required for all worksites (*NSV*_sygd_), the longest (*TR*_zc_) and the shortest times (*TR*_zd_) of a single cycle of all worksites, and the loading (*t*_zc_) and unloading times (*t*_xc_).

If there is only one earliest starting worksite (i.e., *NSE*_gd_ = 1), the number of second earliest starting worksites is *NSC*_gd_ (i.e., *NSC*_gd_ > 0). When *NSC*_gd_ = 1, if the time the truck to return from the earliest starting worksite to the mixing plant is earlier than the starting time of the second earliest worksite, then it is not necessary to correct the departure time of the first truck (it indicates that some trucks have returned to the mixing station before the starting time of second earliest starting worksites). If the *NSV*_bhz_ is greater than the sum of the number of trucks required at the earliest and second earliest starting worksites, then there is no need to adjust the time (it suggests that the number of trucks is sufficient to meet the needs of the trucks at the earliest and second earliest starting worksites). When the *NSV*_bhz_ is insufficient, and the earliest time a truck returns to the mixing station is later than the starting time of the second earliest starting worksites (*TC*_*s*_), it is necessary to advance the departure time by the difference between the *TC* and *TC*_*s*_. When *NSC*_gd_ > 1, the departure time can be adjusted according to the value of *NSC*_gd_. It can be calculated as follows:

11 $$\Delta t = \left\{ {\begin{array}{*{20}l} {0;} \hfill & {\left[ {NSV_{{{\text{bhz}}}} \ge NSV_{{{\text{sygd}}}} } \right] \vee \left[ {\left( {TR_{{{\text{zd}}}} + t_{{{\text{zc}}}} + t_{{{\text{xc}}}} } \right) + \left( {NSV_{{{\text{bhz}}}} - NSC_{{{\text{gd}}}} + 1} \right) \cdot t_{{{\text{zc}}}} < \left( {TC_{{\text{s}}} - TC} \right)} \right]} \hfill \\ {\left( {TR_{{{\text{zd}}}} + t_{{{\text{zc}}}} + t_{{{\text{xc}}}} } \right) - \left( {TC_{{\text{s}}} - TC} \right) + \left( {NSV_{{{\text{bhz}}}} - NSC_{{{\text{gd}}}} + 1} \right) \cdot t_{{{\text{zc}}}} ;} \hfill & {\left[ {NSV_{{{\text{bhz}}}} < NSV_{{{\text{sygd}}}} } \right] \wedge \left[ {\left( {TR_{{{\text{zd}}}} + t_{{{\text{zc}}}} + t_{{{\text{xc}}}} } \right) + \left( {NSV_{{{\text{bhz}}}} - NSC_{{{\text{gd}}}} + 1} \right) \cdot t_{{{\text{zc}}}} \ge \left( {TC_{{\text{s}}} - TC} \right)} \right]} \hfill \\ \end{array} } \right.$$

where, “$\bigwedge$” represents an “and” set.

If there are multiple earliest starting worksites (i.e., *NSE*_gd_ > 1), when *NSC*_gd_ = 1, the departure time can be calculated by Eq. (12); when *NSC*_gd_ > 1, it can be calculated by Eq. (13).

12 $$\Delta t = \left\{ {\begin{array}{*{20}l} {0;} \hfill & {\left( {NSV_{{{\text{sygd}}}} \le NSV_{{{\text{bhz}}}} } \right)} \hfill \\ {\left( {TR_{{{\text{zc}}}} - TR_{{{\text{zd}}}} } \right) + \left( {NSV_{{{\text{bhz}}}} - NSC_{{{\text{gd}}}} + 1} \right) \cdot t_{{{\text{zc}}}} ;} \hfill & {\left( {NSV_{{{\text{sygd}}}} > NSV_{{{\text{bhz}}}} } \right) \wedge \left[ {\left( {TR_{{{\text{zc}}}} - TR_{{{\text{zd}}}} } \right) + \left( {NSV_{{{\text{bhz}}}} - NSE_{{{\text{gd}}}} + 1} \right) \cdot t_{{{\text{zc}}}} - \left( {TC_{{\text{s}}} - TC} \right) \le 0} \right]} \hfill \\ {2 \cdot \left[ {\left( {TR_{{{\text{zc}}}} - TR_{{{\text{zd}}}} } \right) + \left( {NSV_{{{\text{bhz}}}} - NSE_{{{\text{gd}}}} + 1} \right) \cdot t_{{{\text{zc}}}} } \right] - \left( {TC_{{\text{s}}} - TC} \right);} \hfill & {\left( {NSV_{{{\text{sygd}}}} > NSV_{{{\text{bhz}}}} } \right) \wedge \left[ {\left( {TR_{{{\text{zc}}}} - TR_{{{\text{zd}}}} } \right) + \left( {NSV_{{{\text{bhz}}}} - NSE_{{{\text{gd}}}} + 1} \right) \cdot t_{{{\text{zc}}}} - \left( {TC_{{\text{s}}} - TC} \right) > 0} \right]} \hfill \\ \end{array} } \right.$$

13 $$\Delta t = \left\{ {\begin{array}{*{20}l} {0;} \hfill & {\left( {NSV_{{{\text{sygd}}}} \le NSV_{{{\text{bhz}}}} } \right)} \hfill \\ {\left( {TR_{{{\text{zc}}}} - TR_{{{\text{zd}}}} } \right) + \left( {NSV_{{{\text{bhz}}}} - NSE_{{{\text{gd}}}} + 1} \right) \cdot t_{{{\text{zc}}}} + \left( {NSV_{{{\text{bhz}}}} - NSE_{{{\text{gd}}}} - NSC_{{{\text{gd}}}} + 2} \right) \cdot t_{{{\text{zc}}}} ;} \hfill & {\left( {NSV_{{{\text{sygd}}}} > NSV_{{{\text{bhz}}}} } \right) \wedge \left[ {\left( {TR_{{{\text{zc}}}} - TR_{{{\text{zd}}}} } \right) + \left( {NSV_{{{\text{bhz}}}} - NSE_{{{\text{gd}}}} + 1} \right) \cdot t_{{{\text{zc}}}} - \left( {TC_{{\text{s}}} - TC} \right) \le 0} \right]} \hfill \\ {2 \cdot \left[ {\left( {TR_{{{\text{zc}}}} - TR_{{{\text{zd}}}} } \right) + \left( {NSV_{{{\text{bhz}}}} - NSE_{{{\text{gd}}}} + 1} \right) \cdot t_{{{\text{zc}}}} } \right] - \left( {TC_{{\text{s}}} - TC} \right) + \left( {NSV_{{{\text{bhz}}}} - NSE_{{{\text{gd}}}} - NSC_{{{\text{gd}}}} + 1} \right) \cdot t_{{{\text{zc}}}} ;} \hfill & {\left( {NSV_{{{\text{sygd}}}} > NSV_{{{\text{bhz}}}} } \right) \wedge \left[ {\left( {TR_{{{\text{zc}}}} - TR_{{{\text{zd}}}} } \right) + \left( {NSV_{{{\text{bhz}}}} - NSE_{{{\text{gd}}}} + 1} \right) \cdot t_{{{\text{zc}}}} - \left( {TC_{{\text{s}}} - TC} \right) > 0} \right]} \hfill \\ \end{array} } \right.$$

In addition, due to the limitation of the initial setting time of concrete, the maximum waiting time of the truck cannot exceed the given maximum allowable waiting time (*TW*_gc_). Therefore, it is necessary to calculate the correction time (*∆t*′) as follows according to the initial setting time limit.

14 $$\Delta t^{\prime} = \left\{ {\begin{array}{*{20}l} {TW_{{{\text{gc}}}} - t_{{{\text{zc}}}} ;} \hfill & {TC - TR_{{{\text{zd}}}} - T_{{{\text{bhz}}}} - TW_{{{\text{gc}}}} \ge 0} \hfill \\ {TC - TR_{{{\text{zd}}}} - T_{{{\text{bhz}}}} - t_{{{\text{zc}}}} ;} \hfill & {TC - TR_{{{\text{zd}}}} - T_{{{\text{bhz}}}} - TW_{{{\text{gc}}}} < 0} \hfill \\ \end{array} } \right.$$

Finally, compare the correction time (*Δt*) and (*∆t*′), and choose the smaller of the two.

### Departure time of the other trucks

If the truck’s selected worksite has already been dispatched for a truck (namely, this truck is not the first truck to be dispatched to the worksite), the departure time of the truck is calculated according to the number of trucks currently available at the mixing station, as shown in the following equations:

15 $$TI_{m} = \left\{ {\begin{array}{*{20}l} {TI_{m - 1} + t_{{{\text{zc}}}} ;} \hfill & {NSVC_{{{\text{bhz}}}} \ge 1} \hfill \\ {TBF_{m} ;} \hfill & {NSVC_{{{\text{bhz}}}} = 0} \hfill \\ \end{array} } \right.$$

where, ${TI}_{m}$ is the departure time of the *m*-th truck; *TBF*_*m*_ is the soonest time for all trucks in transit returning to the mixing station after *TI*_*m−1*_; *NSVC*_bhz_ is the number of trucks currently available at the mixing station after *TI*_*m−1*_.

If no truck has been dispatched for a selected worksite *i* (namely, this is the first truck to be dispatched to the worksite), it is necessary to estimate the departure time by the following equation based on the starting time of the worksite *i* (*TC*_*i*_), the time required from the mixing station to the worksite (*TRG*_*i*_), and the loading time:

16 $$TI_{m} = TC_{i} - TRG_{i} - t_{{{\text{zc}}}}$$

where, *TRG*_*i*_ is the time required from the mixing station to the worksite (*i* is the worksite selected by the *m*-th truck).

If the result of Eq. (16) is less than the result of Eq. (15) (namely, the regular departure time is later than the departure time estimated based on the start time of the worksite), the departure should be carried out based on the result of Eq. (15). Suppose the result of Eq. (16) is much greater than the result of Eq. (15) (namely, the start time of worksite *i* is very late). In that case, if the departure is carried out according to the result of Eq. (15), the waiting time of the truck is very long, which may exceed the initial setting time of concrete, and the utilization rate of the truck is inadequate. Moreover, if the departure is carried out based on the result of Eq. (16), the subsequent departure will be affected, resulting the truck will wait for the order at the mixing station for a long time, which will cause waste of the truck and affecting other worksites. Therefore, to avoid local optimum or too many invalid solutions, if the result of Eq. (16) is greater than the result of Eq. (15), there is a need to re-select the worksite. According to the time difference ($\Delta {t}_{m}$) between Eqs. (15) and (16), and the average loading time, after extensive trial calculation based on the number of worksites (${NS}_{\text{gd}}$), the number of worksite re-selection (*NAG*) can be calculated by the following equations:

17 $$NAG = \left\langle {\left( {NS_{{{\text{gd}}}} - 1} \right) \cdot \frac{{\vartriangle t_{m} }}{{4 \cdot t_{{{\text{zc}}}} }}} \right\rangle$$

Moreover, this paper compares *Δt*_*m*_ with the possible times after all trucks depart to refine possible departure times.

1. If *Δt*_*m*_ < *t*_zc_, then calculate ${TI}_{m}$ using Eq. (15).
2. Suppose *Δt*_*m*_ ≥ *t*_zc_ and *Δt*_*m*_ < *NS*_gd_∙(0.8∙*t*_*zc*_) + *TMgd*_*i*_, then it is necessary to re-select the worksite according to the procedure described in “Iterative proces”, and then recalculate ${TI}_{m}$ until it reaches the upper limit of the number of recalculations (the number of recalculations is determined by Eq. (17)). If $\Delta {t}_{m}\le {TW}_{\text{gc}}$, then calculate *TI*_*m*_ using Eq. (16); if $\Delta {t}_{m}>{TW}_{\text{gc}}$, then calculate *TI*_*m*_ using Eq. (18).
  18 $$TI_{m} = \left\{ {\begin{array}{*{20}l} {TC_{i} - TRG_{i} - t_{{{\text{zc}}}} ;} \hfill & {\frac{{NSVC_{{{\text{bhz}}}} }}{{NSVgd_{i} }} \ge 1} \hfill \\ {TC_{i} - TRG_{i} - t_{{{\text{zc}}}} - \left\langle {TW_{{{\text{gc}}}} \cdot \frac{{NSVC_{{{\text{bhz}}}} }}{{NSVgd_{i} }}} \right\rangle ;} \hfill & {0.5 \le \frac{{NSVC_{{{\text{bhz}}}} }}{{NSVgd_{i} }} < 1} \hfill \\ {TC_{i} - TRG_{i} - t_{{{\text{zc}}}} - TW_{{{\text{gc}}}} } \hfill & {\frac{{NSVC_{{{\text{bhz}}}} }}{{NSVgd_{i} }} < 0.5} \hfill \\ \end{array} } \right.$$
3. If *Δt*_*m*_ ≥ *t*_*zc*_ and *Δt*_*m*_ ≥ *NS*_gd_∙(0.8∙*t*_zc_) + *TMgd*_*i*_, it is also necessary to re-select the worksite according to the procedure described in “Iterative proces”, and then recalculate ${TI}_{m}$ until it reaches the upper limit of the number of recalculations. If $\Delta {t}_{m}\le {TW}_{\text{gc}}$, then calculate *TI*_*m*_ using Eq. (15); if $\Delta {t}_{m}>{TW}_{\text{gc}}$, then calculate *TI*_*m*_ using Eq. (18).

Since the *NSV*_bhz_ is certain and the trucks can be dispatched multiple times, the time for different trucks to return to the mixing station (*TB*_*j*_) changes where *j* ∈ [1, *NSVC*_bhz_] represents the serial number of different trucks. The updated process is calculated using Algorithm 3.
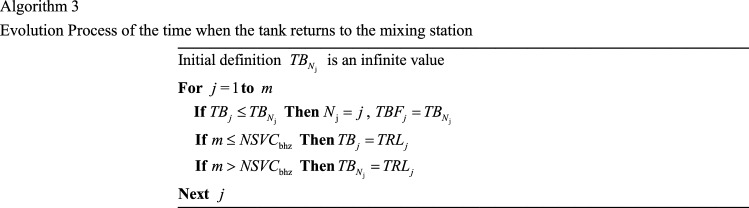


Where, *TRL*_*m*_ is the time when the *m*-th truck returns from the worksite to the mixing station; ${N}_{\text{j}}$ is the serial number of the truck that returns to the mixing station the quickest among all trucks.

The truck number (*j*) is numbered according to the departure sequence. When all trucks depart, the numbering of all trucks is completed. After that, the departure will be arranged according to the quickest return to the mixing station, and the *j* corresponds to it, which is calculated by the following equation.

19 $$j = \left\{ {\begin{array}{*{20}l} {m;} \hfill & {m \le NSVC_{{{\text{bhz}}}} } \hfill \\ {N_{{\text{j}}} ;} \hfill & {m > NSVC_{{{\text{bhz}}}} } \hfill \\ \end{array} } \right.$$

### Determination of waiting time of worksites and trucks

After the ${TI}_{m}$ is determined, the arrival time of the truck to the worksite can be determined based on the time required for the truck to reach the worksite from the mixing station (the time can be calculated according to the real-time data of maps, considering the road conditions, general speed, distance, weather, etc.), which can be shown as follows:

20 $$TA_{m} = TI_{m} + TRG_{i} = TAgc_{i,n}$$

where, *TA*_*m*_ is the time when the *m*-th truck arrives at the worksite from the mixing station; *TAgc*_*i,n*_ is the time when the *n*-th truck arrives at worksite *i*.

When the truck starts pouring, it is necessary to consider whether other trucks are pouring or waiting in line. Therefore, we need to first calculate the waiting time of the truck according to the following equation:

21 $$TD_{m} = \left\{ {\begin{array}{*{20}l} {TLAgc_{i,n - 1} - TAgc_{i,n} ;} \hfill & {NSVA_{i} > 0} \hfill \\ {TC_{i} - TAgc_{i,n} ;} \hfill & {NSVA_{i} = 0} \hfill \\ \end{array} } \right.$$

where, *TD*_*m*_ is the waiting time of the *m*-th truck from the mixing station; *TLAgc*_*i,n−1*_ is the moment when the *n* − 1-th truck leaves worksite *i* after pouring; *NSVA*_*i*_ is the number of trucks arranged for worksite *i*.

If the *TD*_*m*_ is greater than or equal to 0, it means that the truck is waiting while the worksite is not waiting; if the *TD*_*m*_ is less than 0, it means that the truck is not waiting while the worksite is waiting. The corresponding waiting time is calculated as follows:

22 $$\begin{array}{*{20}l} {TDgc_{i,n} = TD_{m} ,TDgd_{i,n} = 0;} \hfill & {TD_{m} \ge 0} \hfill \\ {TDgc_{i,n} = 0,TDgd_{i,n} = - TD_{m} ,TDS_{m} = - TD_{m} ;} \hfill & {TD_{m} < 0} \hfill \\ \end{array}$$

where, *TDS*_*m*_ is the waiting time of the worksite after the *m*-th truck from the mixing station leaves the worksite.

The calculation of the time when the worksite starts pouring, the time when the truck leaves the worksite, and the time when the truck returns to the mixing station is detailed in Eq. (23).

23 $$\begin{gathered} TSAgc_{i,n} = TAgc_{i,n} + TDgc_{i,n} {\kern 1pt} \hfill \\ {\kern 1pt} {\kern 1pt} TLAgc_{i,n} = TSAgc_{i,n} + t_{{{\text{xc}}}} \hfill \\ {\kern 1pt} TRBgc_{i,n} = TLAgc_{i,n} + TRB_{i} \hfill \\ \end{gathered}$$

where, *TSAgc*_*i,n*_ is the moment when the *n*-th truck arrives at worksite *i* and starts pouring; *TRBgc*_*i,n*_ is the moment when the *n*-th truck arrives at worksite *i* and returns to the mixing station; *TRB*_*i*_ is the time required for the truck to return to the mixing station from worksite *i*.

### Correction to the departure time by dual-machine blanking

Moreover, the mixing station is commonly equipped with dual-machine mixing, which can load two trucks simultaneously. The departure time of the truck is corrected according to the vacancy of the mixer. It can be calculated as follows:

24 $$TI^{\prime}_{m} \left\{ {\begin{array}{*{20}l} {TI_{m} ;} \hfill & {\left( {N_{{{\text{mo}}}} = 2} \right){\kern 1pt} \vee \left[ {\left( {TI_{m} - t_{{{\text{zc}}}} - TBF_{m} = 0} \right) \wedge \left( {0 \le N_{{{\text{mo}}}} < 2} \right)} \right]} \hfill \\ {TI_{m} - t_{{{\text{zc}}}} ;} \hfill & {\left( {TI_{m} - TBF_{m} \ne 0} \right) \wedge \left( {0 \le N_{{{\text{mo}}}} < 2} \right)} \hfill \\ \end{array} } \right.$$

where, *TI′*_*m*_ is the departure time of the *m*-th truck from the mixing station after correction; *N*_mo_ is the number of mixers currently occupied.

## Solutions to dynamic dispatching

During the dispatching of concrete, there are often dynamic changes, such as temporary increase or cancellation of orders and an increase or decrease in the number of trucks. In this case, it is necessary to rearrange the static dispatching and form a new dispatching list, while ensuring that the completed orders are not disrupted (including the complete concrete pouring at the worksite and the information of available trucks). Since emergencies are unpredictable, it is difficult and unfeasible to directly build a calculation model for dynamic dispatching. However, by recalling the static calculation model at different times, dynamic dispatching can be easily and quickly achieved.

Based on the static dispatching model, the trucks in the dispatching process are divided into two parts: the first part is the number of trucks available at the mixing station (*NSV1*_bhz_); the second part is the number of trucks that depart from the mixing station but do not return (${NSV2}_{\text{bhz}}$) and the estimated time of each truck returning to the mixing station (*TBS*_*k*_) (k ∈ [1, *NSV2*_bhz_]). When recalling the static dispatching model, the start time of the mixing station (*T*_bhz_) described in “Solutions to static dispatching” should be the current moment (*T*_cur_); the start time of the worksite (*TC*_*i*_) should be the moment when the last truck that have been dispatched to the worksite completes the pouring (*TLAgc*_*i,k*_) (If there is no truck on the way at the worksite, that is, the worksite has not yet started at the moment, then the start time remains unchanged). The total number of trucks required for worksite *i* (*NSVgd*_*i*_) is adjusted according to the actual volume of concrete poured (based on the volume of concrete completely poured by all trucks dispatched to the worksite before the recalculation time). It is calculated as follows:

25 $$NSVgd_{i} = \left\langle {\frac{{VC_{i} - NSVA_{i} \cdot VT}}{VT}} \right\rangle$$

where, *VC*_*i*_ is the total demand of concrete at worksite *i*, dimension L^3^; *VT* is the capacity of the truck, dimension L^3^.

In addition, in the calculation with the static dispatching model, the quickest time for the truck that returns to the mixing station (${TBF}_{m}$) needs to be corrected according to ${TBS}_{k}$ and calculated by Eq. (26).

26 $$TBF_{m} = \min \left\{ {TB_{{N_{{\text{j}}} }} ,TB_{{N_{{\text{k}}} }} } \right\}$$

where, ${TB}_{{N}_{\text{j}}}$ is the soonest time of ${NSV1}_{\text{bhz}}$ trucks available returning to the mixing station; ${TB}_{{N}_{\text{k}}}$ is the soonest time of ${NSV2}_{\text{bhz}}$ trucks available returning to the mixing station. The time when different trucks return to the mixing station (${TB}_{j}$) is updated according to Algorithm 3.

Determination of the *j*: first, determine the number (*NSV3*_bhz_) of trucks returning to the mixing station before the departure time of the earliest worksite according to the return time of the *NSV2*_bhz_ trucks on the way; then, number the *NSV1*_bhz_ + *NSV3*_bhz_ trucks based on the departure sequence; finally, number them sequentially according to the quickest return time of the remaining trucks on the way. The calculations are defined as:

27 $$j = \left\{ {\begin{array}{*{20}l} {m;} \hfill & {m \le \left( {NSV1_{{{\text{bhz}}}} + NSV1_{{{\text{bhz}}}} } \right)} \hfill \\ {N_{{\text{j}}} ;} \hfill & {\left[ {m > \left( {NSV1_{{{\text{bhz}}}} + NSV1_{{{\text{bhz}}}} } \right)} \right] \wedge \left( {TB_{{N_{{\text{j}}} }} \le TB_{{N_{{\text{k}}} }} } \right)} \hfill \\ {N_{{\text{k}}} ;} \hfill & {\left[ {m > \left( {NSV1_{{{\text{bhz}}}} + NSV1_{{{\text{bhz}}}} } \right)} \right] \wedge \left( {TB_{{N_{{\text{j}}} }} > TB_{{N_{{\text{k}}} }} } \right)} \hfill \\ \end{array} } \right.$$

Regarding the number of trucks currently available at the mixing station (*NSVC*_bhz_), for each truck sent out from the mixing station, there is one less truck available at the mixing station; conversely, for each truck returning to the mixing station, there is one more truck available at the mixing station. Additionally, *NSVC*_bhz_ needs to be corrected according to *TBS*_*k*_. See Algorithm 4 for the specific calculation process.
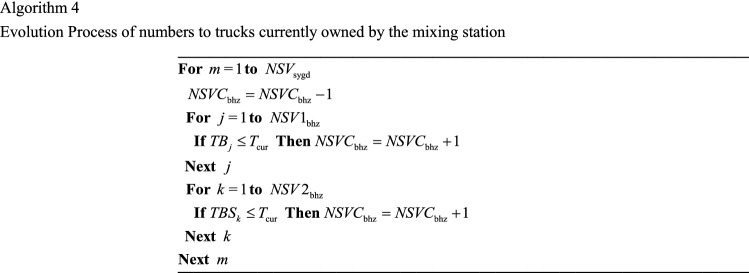


## Calculating example analysis

Suppose three worksites require concrete, namely, A, B, and C. The corresponding parameters are shown in Table 2.

**Table 2:** Corresponding calculation parameters of each worksites.

Name of worksites	$i$	${VC}_{i}$ (${\text{m}}^{3}$)	${TRG}_{i}$ (min)	${TRB}_{i}$ (min)	${TC}_{i}$	${TMgd}_{i}$
A	1	100	26	15	8:10	60
B	2	130	21	18	9:10	60
C	3	130	32	23	11:10	40

There are currently 10 trucks at the mixing station, with a tank capacity of 8 m^3^. The average loading time of the mixing station is 5 min and the average unloading time is 10 min. In the calculation, the individual perceived weight (${c}_{1}$) and the overall social weight (${c}_{2}$) are taken as 2.5 and 1.45, respectively; the particle swarm size is taken as 50, and the number of iterations is 600; the mixing station is equipped with dual-machine to loading the truck; the start time of the mixing station is 07:30 a.m.

The calculation results are shown in Table 3. The cumulative waiting time of all worksites (*TS*_gd_) is 73 min, the longest waiting time of worksites is 24 min (the 18th departure), and the cumulative waiting time of all trucks (*TS*_gc_) is 473 min; the longest waiting time of worksite A (*TMgd*_*i*_) is 20 min and the longest waiting time of the truck (*TMgc*_*i*_) is 30 min, *TMgd*_*i*_ is 24 min and *TMgc*_*i*_ is 23 min for worksite B, *TMgd*_*i*_ is 10 min and *TMgc*_*i*_ is 26 min for worksite C. All the above meet the construction requirements.

**Table 3:** The calculation results.

*m*	*i*	${TI}_{m}$	${TA}_{m}$	${TD}_{m}$	${TLAgc}_{i,n}$	${TRBgc}_{i,n}$	$j$
1	A	7:39	8:10	0	8:20	8:35	1
2	A	7:39	8:10	10	8:30	8:45	2
3	A	7:44	8:15	15	8:40	8:55	3
4	A	7:44	8:15	25	8:50	9:05	4
5	A	7:49	8:20	30	9:0	9:15	5
6	B	8:44	9:10	0	9:20	9:38	6
7	B	8:44	9:10	10	9:30	9:48	7
8	A	8:49	9:20	− 20	9:30	9:45	8
9	A	8:49	9:20	10	9:40	9:55	9
10	A	8:54	9:25	15	9:50	10:05	10
11	B	8:54	9:20	10	9:40	9:58	1
12	A	8:59	9:30	20	10:0	10:15	2
13	A	8:59	9:30	30	10:10	10:25	3
14	B	9:04	9:30	10	9:50	10:08	4
15	A	9:15	9:46	24	10:20	10:35	5
16	A	9:38	10:09	11	10:30	10:45	6
17	A (4)	9:45	10:16	14	10:40	10:55	8
18	B	9:48	10:14	− 24	10:24	10:42	7
19	B	9:55	10:21	3	10:34	10:52	9
20	B	9:58	10:24	10	10:44	11:02	1
21	B	10:05	10:31	13	10:54	11:12	10
22	B	10:08	10:34	20	11:4	11:22	4
23	B	10:15	10:41	23	11:14	11:32	2
24	B	10:25	10:51	23	11:24	11:42	3
25	C	10:35	11:12	− 2	11:22	11:45	5
26	C	10:42	11:19	3	11:32	11:55	7
27	C	10:45	11:22	10	11:42	12:05	6
28	C	10:52	11:29	13	11:52	12:15	9
29	B	10:55	11:21	3	11:34	11:52	8
30	B	11:02	11:28	6	11:44	12:02	1
31	C	11:12	11:49	3	12:2	12:25	10
32	C	11:22	11:59	3	12:12	12:35	4
33	B	11:32	11:58	− 14	12:8	12:26	2
34	B	11:42	12:08	0	12:18	12:36	3
35	C	11:45	12:22	− 10	12:32	12:55	5
36	C	11:52	12:29	3	12:42	13:05	8
37	B	11:55	12:21	− 3	12:31	12:49	7
38	B (6)	12:02	12:28	3	12:41	12:59	1
39	C	12:05	12:42	0	12:52	13:15	6
40	C	12:15	12:52	0	13:2	13:25	9
41	C	12:25	13:02	0	13:12	13:35	10
42	C	12:26	13:03	9	13:22	13:45	2
43	C	12:35	13:12	10	13:32	13:55	4
44	C	12:36	13:13	19	13:42	14:05	3
45	C	12:49	13:26	16	13:52	14:15	7
46	C	12:55	13:32	20	14:2	14:25	5
47	C (6)	12:59	13:36	26	14:12	14:35	1

Judging from the departure time (*TI*_*m*_), when the mixing station has sufficient trucks, the departure time is every 5 min; when the start time of the selected worksite is late (*m* = 6), the model (see “Departure time of the first truck”) will automatically match a more appropriate departure time rather than 7:54 a.m. (otherwise, it will increase the waiting time of the truck); similarly, when a different worksite is selected and the truck is dispatched for this worksite for the first time (*m* = 6, 25), the departure time is also corrected and adjusted according to the start time of the worksite (see “Departure time of the first truck”). When there is no available truck at the mixing station (such as after the 10th departure), the optimization model will determine the departure time based on the time when the truck returns to the mixing station (see “Departure time of the other trucks”). For example, when Truck 8 (*m* = 17, *j* = 8) returns to the mixing station earlier than Truck 7 (*m* = 18, *j* = 8), Truck 10 will be dispatched for the 17th departure and the departure time is the same as the return time to the mixing station. Similarly, the 20th and 21st departures are also arranged based on the time when the truck returns to the mixing station. The calculation time of the program is 18.5 s.

Assuming that at 9:00 a.m., the concrete demand of worksite A is adjusted to 116 and worksite B temporarily cancels, then the dispatching at this time needs to consider the tasks that have been completed before 9:00 a.m. According to Tables 2 and 3, a total of 10 trucks are arranged to worksite A before 9:00 a.m., and the time when the pouring is completed is 10:10 (*m* = 13), and the pouring volume is 80 m^3^. Then, the start time of worksite A is adjusted to 10:10 a.m., and the demand quantity is 36 m^3^. Since worksite C has not yet started, adjustments may not need to be made for this worksite. At this time, the number of available trucks at the mixing station (*NSV1*_bhz_) is 0, and *NSV2*_bhz_ = 10, and the return time to the mixing station is shown as ${TRBgc}_{i,n}$ (*m* = 4–13) in Table 3. The start time of the mixing station is adjusted to the current time 9:00 a.m. The model described in “Solutions to dynamic dispatching” is used to calculate the parameters, and the results are shown in Table 4. The number *j'* corresponds to *j* in Table 3. The calculation time of the program is 9.2 s.

**Table 4:** The calculation results after order adjustment.

*m*	*i*	${TI}_{m}$	${TA}_{m}$	${TD}_{m}$	${TLAgc}_{i,n}$	${TRBgc}_{i,n}$	$j$	*j*′
1	A	9:39	10:10	0	10:20	10:35	1	4 (*m* = 4)
2	A	9:39	10:10	10	10:30	10:45	2	5 (*m* = 5)
3	A	9:44	10:15	15	10:40	10:55	3	6 (*m* = 6)
4	A	9:45	10:16	24	10:50	11:05	9	8 (*m* = 8)
5	A (4)	9:45	10:16	34	11:00	11:15	10	7 (*m* = 7)
6	C	10:33	11:10	0	11:20	11:43	8	9 (*m* = 9)
7	C	10:33	11:10	10	11:30	11:53	6	1 (*m* = 11)
8	C	10:38	11:15	15	11:40	12:03	7	10 (*m* = 10)
9	C	10:38	11:15	25	11:50	12:13	5	2 (*m* = 12)
10	C	10:43	11:20	30	12:00	12:23	4	3 (*m* = 13)
11	C	10:43	11:20	40	12:10	12:33	1	–
12	C	10:48	11:25	45	12:20	12:43	2	–
13	C	10:55	11:32	48	12:30	12:53	3	–
14	C	11:05	11:42	48	12:40	13:03	9	–
15	C	11:15	11:52	48	12:50	13:13	10	–
16	C	11:43	12:20	30	13:0	13:23	8	–
17	C	11:53	12:30	30	13:10	13:33	6	–
18	C	12:03	12:40	30	13:20	13:43	7	–
19	C	12:13	12:50	30	13:30	13:53	5	–
20	C	12:23	13:0	30	13:40	14:03	4	–
21	C	12:33	13:10	30	13:50	14:13	1	–
22	C (6)	12:43	13:20	30	14:00	14:23	2	–

The calculation results show that dynamic dispatching (“Solutions to dynamic dispatching”) can realize the rescheduling without affecting the previously completed tasks. According to Eq. (27), three trucks have returned to the mixing station before 9:39 a.m. at the departure time of the earliest worksite (*m* = 4–6 in Table 4). Therefore, in the first three departures (*m* = 1–3), the truck number is the same as the departure number, while the departure number of the remaining seven trucks is related to the given return time to the mixing station. The cumulative waiting time of all worksites (*TS*_gd_) is 0 min, and the cumulative waiting time of all trucks (*TS*_gc_) is 602 min, indicating that the number of trucks is sufficient.

Based on the above situation, suppose that trucks with truck numbers 1, 2, 3, and 10 will no longer be assigned tasks after completing the task (e.g., the number of trucks is adjusted from 10 to 6), then the calculation results are shown in Table 5. The number *j'* corresponds to *j* in Table 3. The calculation time of the program is 8.2 s.

**Table 5:** The calculation results after the order and the number of trucks are adjusted.

*m*	*i*	${TI}_{m}$	${TA}_{m}$	${TD}_{m}$	${TLAgc}_{i,n}$	${TRBgc}_{i,n}$	${N}_{\text{v}}$	*N*′*v*
1	A	9:39	10:10	0	10:20	10:35	1	4 (*m* = 4)
2	A	9:39	10:10	10	10:30	10:45	2	5 (*m* = 5)
3	A	9:44	10:15	15	10:40	10:55	3	6 (*m* = 6)
4	A	9:45	10:16	24	10:50	11:05	5	8 (*m* = 8)
5	A (4)	9:45	10:16	34	11:00	11:15	6	7 (*m* = 7)
6	C	10:33	11:10	0	11:20	11:43	4	9 (*m* = 9)
7	C	10:35	11:12	8	11:30	11:53	1	–
8	C	10:45	11:22	8	11:40	12:03	2	–
9	C	10:55	11:32	8	11:50	12:13	3	–
10	C	11:05	11:42	8	12:0	12:23	5	–
11	C	11:15	11:52	8	12:10	12:33	6	–
12	C	11:43	12:20	− 10	12:30	12:53	4	–
13	C	11:53	12:30	0	12:40	13:03	1	–
14	C	12:03	12:40	0	12:50	13:13	2	–
15	C	12:13	12:50	0	13:0	13:23	3	–
16	C	12:23	13:0	0	13:10	13:33	5	–
17	C	12:33	13:10	0	13:20	13:43	6	–
18	C	12:53	13:30	− 10	13:40	14:03	4	–
19	C	13:03	13:40	0	13:50	14:13	1	–
20	C	13:13	13:50	0	14:0	14:23	2	–
21	C	13:23	14:00	0	14:10	14:33	3	–
22	C (6)	13:33	14:10	0	14:20	14:43	5	–

The cumulative waiting time of all worksites (*TS*_gd_) is 20 min, and the cumulative waiting time of all trucks (*TS*_gc_) is 123 min, suggesting that the number of trucks is sufficient. Comparing Table 5 and Table 4, after the number of trucks is reduced, the waiting time of trucks decrease significantly, while the waiting time of worksites increases accordingly; the first six departure times are consistent with the results in Table 4; for the 7th departure, the departure time is the same as the quickest return time to the mixing station (*m* = 1) because there is no available truck at the mixing station. In addition, with the reduction in the number of trucks, the overall time to complete is the time when the *n* − 1-th truck leaves worksite *i* after pour task at all worksites increases by 20 min (e.g., the completion time in Table 5 is 14: 20 p.m. and the completion time in Table 4 is 14:00 p.m.).

## Conclusions

This paper builds a theoretical model for intelligent dispatching of transport vehicles by reviewing the existing literature and actual engineering data and exploring a particle swarm optimization promotion strategy suitable for the dynamic dispatching of transport vehicles in linear engineering projects. This model improves the inertia weight and boundary processing. It also elaborates on the initialization and iterative process, and the improvements in the process are more suitable for actual problems. In addition, the paper analyzes various influencing factors for different waiting times, considering the actual problem's requirements. It proposes a new objective function in the dispatching algorithm that dramatically improves particle swarm convergence speed and minimizes the influence of local optimum on global optimum.

Taking into consideration factors such as the starting time of the mixing station, the loading and unloading time of the truck, the starting time of the worksite, the number of trucks required, and the time required for the truck to cycle once, this study also makes fine-tuned adjustments to the various parameters in the calculation process, making the calculation result more accurate. In addition, the dispatching for dynamic changes such as adding or canceling orders, increasing or reducing the number of available trucks can be conveniently realized, and the situation of dual-machine blanking at the mixing station is also considered.

This study provides an overall solution supported by the new generation of information technology for the concrete transportation dispatching management of the engineering project, and will greatly improve the economic benefits of mixing station and concrete transport vehicles.

## Data Availability

All data generated and analysed during this study are included in this published article.

## Abbreviations

*c*_*1*_, *c*_*2*_: Individual perceived weights and overall social weights
${{\varvec{g}}{\varvec{b}}{\varvec{e}}{\varvec{s}}{\varvec{t}}}^{{\varvec{t}}}$: Global optimal of all particles
*i*: Sequence number of all worksites sorted
*j*: Truck number, *j* ∈ [$$1,{NSVC}_{\text{bhz}}]$$
$k$: Number of the truck issued by the mixing station that has not returned to the mixing station, $k\in \left[1,{NSV2}_{\text{bhz}}\right]$
*m*: Cumulative number of trucks delivered by the mixing station
*N*_gc_: Total number of departures required to complete the task
${N}_{\text{k}}$: Among the ${NSV2}_{\text{bhz}}$ trucks on the way, number of the soonest truck returning to the mixing station
${N}_{\text{j}}$: Serial number of the truck that returns to the mixing station the quickest among all trucks
${N}_{\text{mo}}$: Current number of mixers occupied
$NAG$: Number of worksite re-selection
${NS}_{\text{gd}}$: Number of worksites
*NSC*_gd_: Number of second earliest starting worksites
*NSE*_gd_: Number of earliest worksite
*NSV*_bhz_: Total number of trucks available in the mixing station
*NSV*_sygd_: Total number of trucks required for all worksites
${NSV1}_{\text{bhz}}$: Current iteration step *t*, number of trucks available at the mixing station
${NSV2}_{\text{bhz}}$: Current iteration step *t*, number of trucks that depart from the mixing station but do not return
${NSV3}_{\text{bhz}}$: Among the ${NSV2}_{\text{bhz}}$ trucks on the way, the number of trucks returning to the mixing station before the departure time of the earliest worksite
${NSVA}_{i}$: Number of trucks arranged for worksite *i*
${NSVC}_{\text{bhz}}$: Number of trucks currently available at the mixing station after *TI*_*m-1*_
${NSVgd}_{i}$: Total number of trucks required for worksite *i*
*p*: Population size
${{\varvec{p}}{\varvec{b}}{\varvec{e}}{\varvec{s}}{\varvec{t}}}_{p}^{t}$: Individual optimal of particle *i* of the *t*^*th*^ generation
***r***_*1*_, ***r***_*2*_: Two random *D*-dimensional vector parameters evenly distributed in [0, 1]
*t*: Number of iterations of the current calculation step
*t*_ar_: The shortest time required for a single cycle of the truck at the earliest worksite
${t}_{\text{max}}$: Maximum number of iterations
*t*_xc_: Time required to unload the truck
*t*_zc_: Time required to load the truck
${T}_{\text{cur}}$: Current time at which the algorithm is recalled for calculation
*T*_bhz_: Starting time of the mixing station
${TA}_{m}$: Time when the *m*-th truck arrives at the worksite from the mixing station
${TAgc}_{i,n}$: Time when the *n*-th truck arrives at worksite *i*.
${TB}_{j}$: Time when different tanks return to the mixing station
${TBF}_{m}$: The soonest time for all trucks in transit returning to the mixing station after *TI*_*m*__*−*__*1*_
${TBS}_{k}$: Estimated time of each vehicle returning to the mixing station
*TC*: Starting time of the earliest worksite
${TC}_{i}$: Starting time of the worksite *i*
*TC*_s_: Starting time of second earliest starting worksites
${TD}_{m}$: Waiting time of the *m*-th truck from the mixing station
${TDgc}_{i,n}$: Waiting time of the *n*-th truck leaving the worksite *i*
${TDgd}_{i,n}$: Waiting time of the worksite when the *n*-th truck leaves the worksite *i*.
${TDS}_{m}$: Waiting time of the worksite after the *m*-th truck from the mixing station leaves the worksite
${Tgd}_{i}$, ${Tgc}_{i}$: Total waiting time of worksite and truck
${TI}_{1}$: Departure time of the first truck
${TI}_{m}$: Departure time of the *m*-th truck
*TI*′_*m*_: Departure time of the *m*-th truck from the mixing station after correction
${TLAgc}_{i,n-1}$: Time when the *n* − 1-th truck leaves worksite *i* after is the time when the n − 1-th truck leaves worksite i after pouring
${TMgc}_{i}$: The longest waiting time for all trucks at worksite *i*
${TMgd}_{i}$: The longest waiting time for all worksites at worksite *i*
*TR*_zc_, *TR*_zd_: The longest and the shortest times of a single cycle of all worksites
${TRB}_{i}$: Time required for the truck to return to the mixing station from worksite *i*
${TRBgc}_{i,n}$: Time when the *n*-th truck arrives at worksite *i* and returns to the mixing station
${TRG}_{i}$: Time required from the mixing station to the worksite
${TRL}_{m}$: Time when the *m*-th truck returns from the worksite to the mixing station
${TS}_{\text{gd}}$, ${TS}_{\text{gc}}$: Cumulative waiting time of all worksites and trucks
${TSAgc}_{i,n}$: Time when the *n*-th truck arrives at worksite *i* and starts pouring
*TW*_gc_: The given maximum allowable waiting time for truck
${{\varvec{v}}}_{p}^{t}$, ${{\varvec{x}}}_{p}^{t}$: Velocity and position and direction vectors of particle *i* in the *t*-th generation
${VC}_{i}$: Total demand of concrete at worksite *i*, ${\text{L}}^{3}$
$VT$: Capacity of the truck, ${\text{L}}^{3}$
*w*: Weight of inertia
${w}_{\text{max}}$: The maximum value of *w*, and 0.9 is recommended
${w}_{\text{min}}$: The minimum value of w*,* and 0.4 is recommended
${x}_{1}$: Sequence number of the worksite selected for the first departure
${x}_{{N}_{\text{gc}}}$: Sequence number of the worksite selected for the last departure
${x}_{\text{max}}$: The maximum value for the sequence number of the worksite
$\Delta t$: Time difference corrected by factors such as the time and number of earliest starting worksites and second earliest starting worksites and the maximum
*∆t′*: Correction time of $\Delta t$
$\Delta {t}_{m}$: Time difference between Eqs. (15) and (16)

## Special calculation symbols

<>: Rounding to an integer
$\bigvee$: An “or” set.
$\bigwedge$: An “and” set
*Rnd*: A random number in [0, 1]
